# Supplementary material for: Converging deep learning and human-observed tumor-adipocyte interaction as a biomarker in colorectal cancer
Source: Commun Med (Lond). 2024 Aug 15;4:163. doi: 10.1038/s43856-024-00589-6 (PMC11327259; doi:10.1038/s43856-024-00589-6)
Supplement: Supplementary file 2 — Supplementary Material [file 43856_2024_589_MOESM2_ESM.pdf]

Supplementary Material for

Converging deep learning and human-observed tumor-adipocyte interaction as a biomarker in colorectal cancer

Nic G. Reitsam*[1,2], MD; Bianca Grosser[1,2], MD; David F. Steiner[3], MD, PhD; Veselin Grozdanov[4], PhD; Ellery Wulczyn[3], MS; Vincenzo L'Imperio[5], MD; Markus Plass[6], MSc; Heimo Müller[6], PhD; Kurt Zatloukal[6], MD, Prof; Hannah S. Muti[7,8], MD; Jakob Nikolas Kather[7,9,10,11], MD, MSc, Prof; Bruno Märkl[1,2], MD, Prof.

[1]Pathology, Medical Faculty, University of Augsburg, Augsburg, Germany.
[2]Bavarian Cancer Research Center (BZKF), Augsburg, Germany
[3]Google Health, Google LLC, Palo Alto, California
[4]Department of Neurology, Ulm University, Ulm, Germany
[5]Department of Medicine and Surgery, Pathology, University of Milano-Bicocca, IRCCS (Scientific Institute for Research, Hospitalization and Healthcare) Fondazione San Gerardo dei Tintori, Monza, Italy
[6]Medical University of Graz, Diagnostic and Research Institute of Pathology, Graz, Austria
[7]Else Kroener Fresenius Center for Digital Health, Technical University Dresden, Dresden, Germany
[8]Department of Visceral, Thoracic and Vascular Surgery, University Hospital Carl Gustav Carus Dresden, Dresden, Germany
[9]Pathology & Data Analytics, Leeds Institute of Medical Research at St James's, University of Leeds, Leeds, United Kingdom
[10]Department of Medicine I, University Hospital Dresden, Dresden, Germany
[11]Medical Oncology, National Center for Tumor Diseases (NCT), University Hospital Heidelberg, Heidelberg, Germany

*Corresponding Author:
Dr. med. Nic Gabriel Reitsam
Institute of Pathology and Molecular Diagnostics
University Hospital Augsburg
86150 Germany
nic.reitsam@uka-science.de, nic.reitsam@uk-augsburg.de
ORCID: https://orcid.org/0000-0002-0070-3158

a

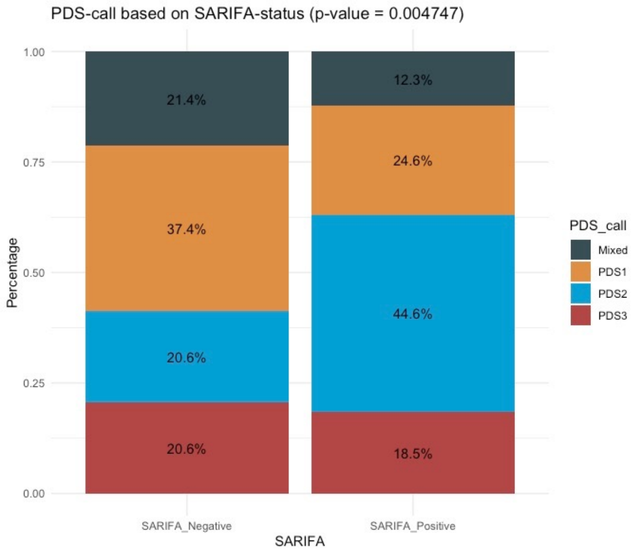

Pathway-derived Subtypes

For more Information refer to Dunne & Arends, Virchows Archive 2024

PDS1: Fast-cycling and MYC activation
PDS2: Immune, Stroma, EMT, TGF-beta
PDS3: slow-cycling repressed for cancer hallmarks, epithelial differentiation

b

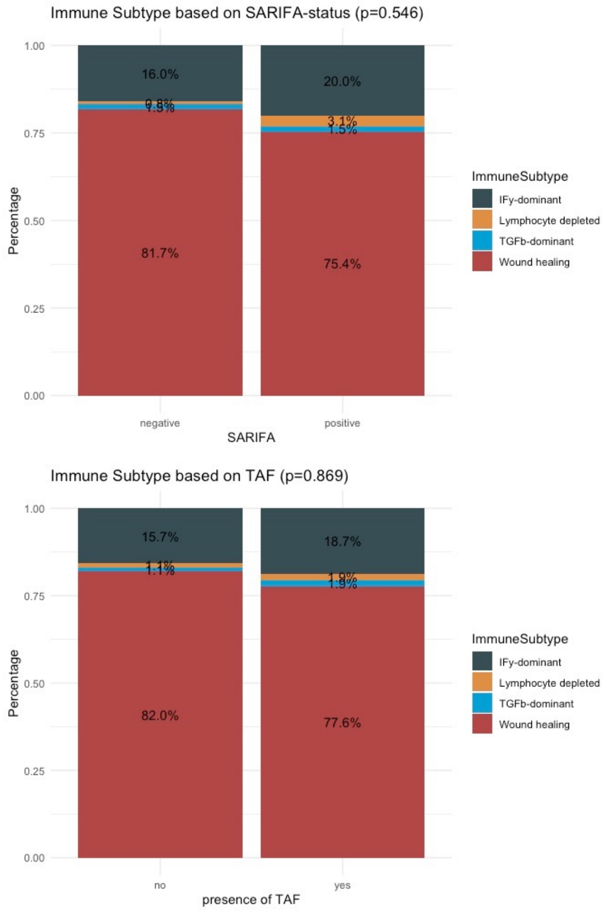

Figure S1. PDS-calls and pan-cancer IS split by SARIFA-status or presence of TAF. a. SARIFA-positive CRCs show statistically different PDS-calls[1], with an enrichment of PDS2 among SARIFA-positive CRCs (p=0.004747, chi-squared test). **b.** Pan-cancer IS[2] does not show any significant SARIFA- or TAF-dependent differences (each p>0.05, chi-squared test), with most CRC cases in general being of '*interferon-gamma dominant*' or '*wound healing subtype'*.
For detailed information on the background of PDS refer to Dunne & Arends[3].
Normalized counts for n=196 samples (TCGA-CRC) were used as previously described[4].
CRC: colorectal cancer, IS: immune subtyping, PDS: pathway-derived subtyping, SARIFA: Stroma AReactive Invasion Front Areas, TAF: tumor adipose feature.

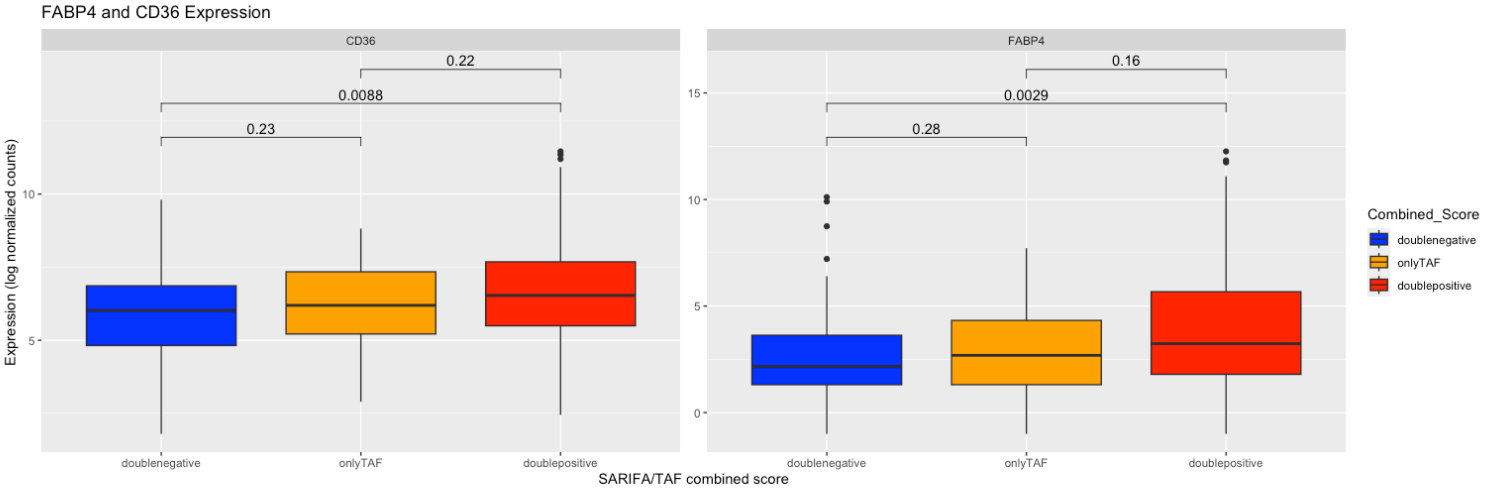

Figure S2. FABP4 and CD36 Expression based on a combined SARIFA/TAF score. SARIFA-positive/TAF-present (*double-positive*) CRCs show a significantly increased expression of *CD36* and *FABP4* compared to SARIFA-negative/TAF-absent (*double-negative*) CRCs (each p-value ≤ 0.01). CRCs with TAF but SARIFA-negativity (*only TAF*) show a trend to a higher expression than SARIFA-negative/TAF-absent CRCs, and a trend-wise lower expression than SARIFA-positive/TAF-present CRCs of *FABP4* and *CD36* – however, without reaching statistical significance (each p-value above 0.05).

Normalized counts for n=196 samples (TCGA-CRC) were used as previously described[4].

Double negativity: SARIFA-negative and no TAF; only TAF: SARIFA-negativity but TAF; double positivity: SARIFA-positive and (logically) with presence of TAF.

Boxplots depict median and interquartile range.

CRC: colorectal cancer, CD36: fatty-acid translocase, FABP4: fatty-acid binding protein 4, SARIFA: Stroma AReactive Invasion Front Areas, TAF: tumor adipose feature.

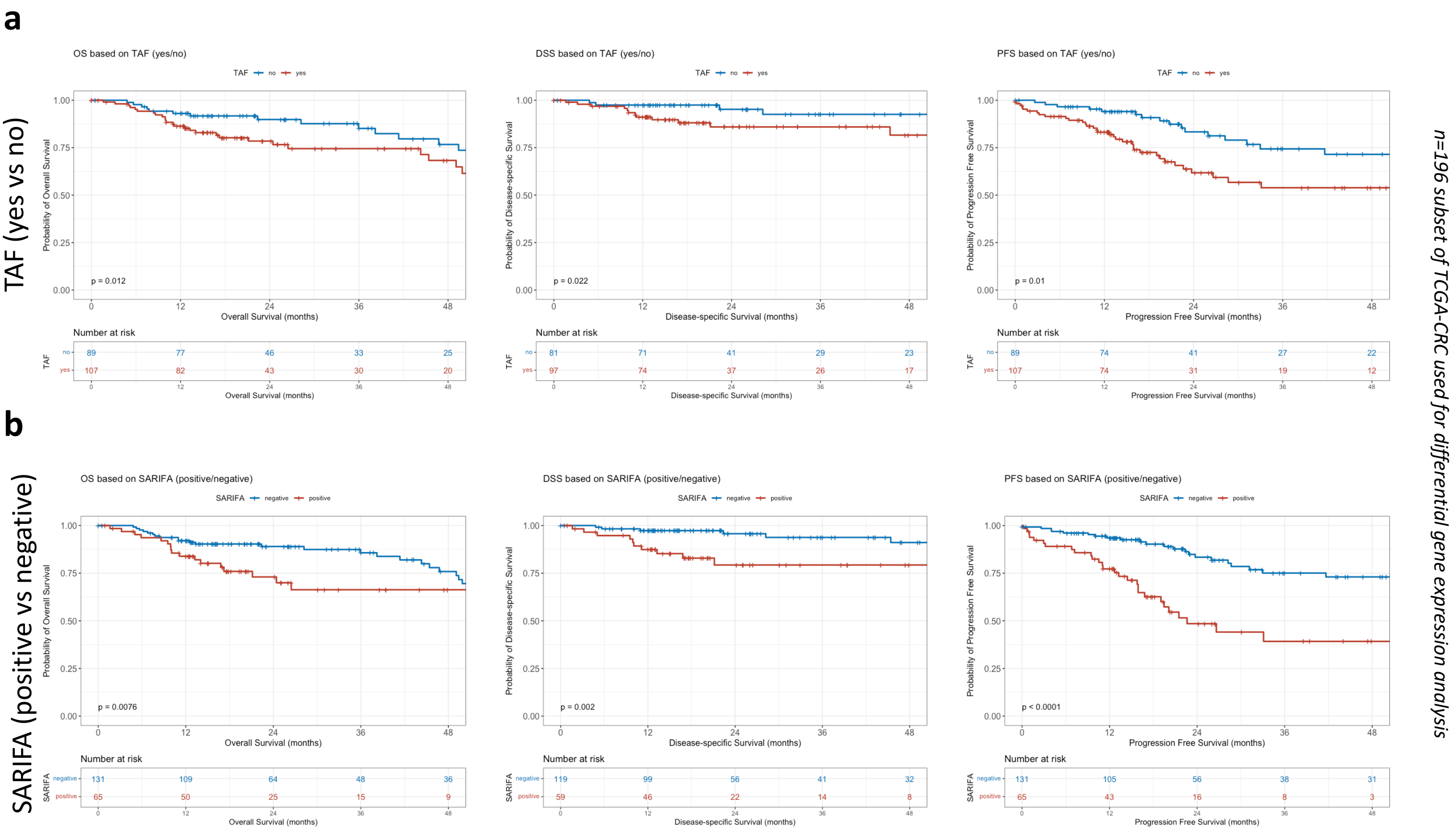

Figure S3. Survival analysis stratified by TAF (yes/no) and SARIFA (positive/negative) a. Kaplan-Meier curves based on TAF (yes/no) for OS, DSS, and PFS, showing significant worse outcomes of patients with CRCs with TAF (each p<0.05, log-rank test). **b.** Kaplan-Meier curves based on SARIFA-status for OS, DSS, PFS, showing poor survival outcomes for SARIFA-positive CRC patients (each p<0.05, log-rank test).

Note that here only the 196 samples[4] of TCGA-CRC used for differential gene expression analysis were included as also the survival curves based on *FABP4* and *CD36* expression and stratified by SARIFA and TAF were based on this subset.

CD36: fatty-acid translocase, CRC: colorectal cancer, DSS: disease-specific survival, FABP4: fatty-acid binding protein 4, PFS: progression free survival, OS: overall survival. TAF: tumor adipose feature.

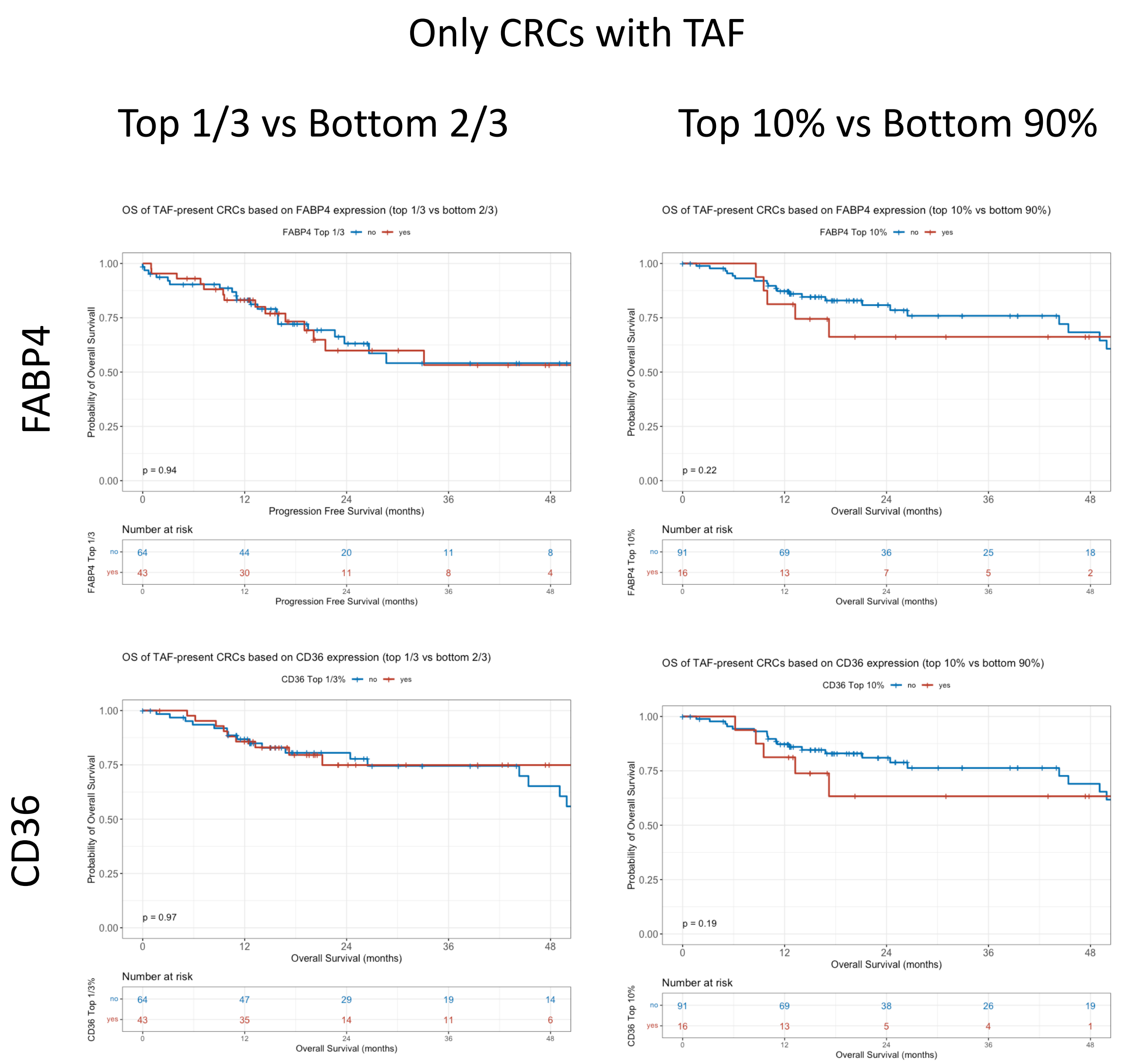

Figure S4. Survival analysis only of CRCs with TAF stratified by *FABP4* and *CD36.* No significant survival differences could be observed when stratifying CRCs with TAF further by *FABP4* and *CD36* expression with regards to OS (each p>0.05, log-rank test). Please refer to Table S4 for the additional stratified analysis.

Note that here only the 196 samples[4] of TCGA-CRC used for differential gene expression analysis were included.

CD36: fatty-acid translocase, CRC: colorectal cancer, FABP4: fatty-acid binding protein 4, OS: overall survival, TAF: tumor adipose feature.

Table S1. Interobserver agreement of overlap

SARIFA/TAF_similiar_feature	*Overlap Pathologist 2 (bg)*	
Overlap Pathologist1 (nr)	***no***	**yes**
**no**	78	8
**yes**	6	128
**Measure of Agreement: Kappa = 0.87**		

Table S2. Reasons for discrepancies in *L'Imperio et al*. and *Krogue et al.*

Patches	*Reasons for misssing overlap with SARIFA*
L'Imperio et al. 2023[5]	
Figure 1: Examples of TAF	Complete overlap between SARIFA/TAF
Supplementary Online Content	1 case (eFigure 4B) in which desmoplastic stroma is between adipocytes and tumor cells at the invasion front; thus no direct tumor-adipocyte interaction here, which is required for SARIFA-positivity. However, it is only a small rim of desmoplastic stroma (close!).
Krogue et al. 2023[5]	
Figure 2: Temporal Validation	Only adipocytes, connective tissue and inflammatory cells are depicted - but no tumor cells. SARIFA-positivity requires direct tumor-adipocyte interaction.
Figure 2: External Validation 1a	In 1 patch no direct-tumor adipocyte interaction due to intervening stroma, and in 1 patch only adipocytes and connective tissue without tumor cells. SARIFA-positivity requires direct tumor-adipocyte interaction.
Supplementary Figure S2	1) only adipose and connective tissue/smooth muscle cells ± inflammatory cells without tumor cells<br>2) no direct tumor-adipocyte interaction<br>3) only mucin without viable tumor cells close to adipocytes (also no direct contact)<br>4) no tumor cells depicted (normal epithelium with inflammatory infiltrate)

Table S3. Differential gene expression analysis results of selected genes associated with lipid metabolism: SARIFA-positive/TAF-present (double positive) vs SARIFA-negative/TAF-present (only TAF)

Negative LFC indicates upregulation in SARIFA-present/TAF-present (double positive)

Gene	baseMean	log2FoldChange	lfcSE	stat	pvalue	padj
FABP4	127.822194	-4.2648266	0.53819915	-7.9242537	2.30E-15	2.09E-11
PLIN1	26.3713833	-2.4940085	0.36727632	-6.7905507	1.12E-11	6.79E-08
ADIPOQ	65.5843555	-5.9623776	1.13678584	-5.2449435	1.56E-07	0.00021913
PLIN4	227.286135	-2.4203509	0.46468505	-5.2085836	1.90E-07	0.00024767
CD36	175.725187	-1.4981494	0.31321216	-4.7831775	1.73E-06	0.00136701

Table S4. Survival analysis stratifedy by TAF and SARIFA, as well as *CD36* and *FABP4* expression

Subgroup	Gene	Comparison	Endpoint	p-value (log-rank test)
**SARIFA-positive**	*FABP4*	top 1/3 vs bottom 2/3	PFS	0.96
	FABP4	top 1/3 vs bottom 2/3	OS	0.26
	FABP4	top 10% vs bottom 90%	PFS	0.32
	FABP4	top 10% vs bottom 90%	OS	0.27
	CD36	top 1/3 vs bottom 2/3	PFS	0.11
	CD36	top 1/3 vs bottom 2/3	OS	0.41
	CD36	top 10% vs bottom 90%	PFS	0.4
	CD36	top 10% vs bottom 90%	OS	0.41
**SARIFA-negative**	*FABP4*	top 1/3 vs bottom 2/3	PFS	0.42
	FABP4	top 1/3 vs bottom 2/3	OS	0.097
	FABP4	top 10% vs bottom 90%	PFS	0.19
	FABP4	top 10% vs bottom 90%	OS	0.23
	CD36	top 1/3 vs bottom 2/3	PFS	0.53
	CD36	top 1/3 vs bottom 2/3	OS	0.36
	CD36	top 10% vs bottom 90%	PFS	0.58
	CD36	top 10% vs bottom 90%	OS	0.96
**TAF-present**	*FABP4*	top 1/3 vs bottom 2/3	PFS	0.94
	FABP4	top 1/3 vs bottom 2/3	OS	0.52
	FABP4	top 10% vs bottom 90%	PFS	0.79
	FABP4	top 10% vs bottom 90%	OS	0.22
	CD36	top 1/3 vs bottom 2/3	PFS	0.36
	CD36	top 1/3 vs bottom 2/3	OS	0.97
	CD36	top 10% vs bottom 90%	PFS	0.66
	CD36	top 10% vs bottom 90%	OS	0.19
**TAF-absent**	*FABP4*	top 1/3 vs bottom 2/3	PFS	0.15
	FABP4	top 1/3 vs bottom 2/3	OS	0.094
	FABP4	top 10% vs bottom 90%	PFS	0.092
	FABP4	top 10% vs bottom 90%	OS	0.28
	CD36	top 1/3 vs bottom 2/3	PFS	0.59
	CD36	top 1/3 vs bottom 2/3	OS	0.065
	CD36	top 10% vs bottom 90%	PFS	0.29
	CD36	top 10% vs bottom 90%	OS	0.38

OS: overall survival, PFS: progression free survival, SARIFA: Stroma AReactive Invasion Front Areas, TAF: tumor adipose feature

References (Supplementary Material)

1. Malla, S. B. *et al.* Pathway level subtyping identifies a slow-cycling biological phenotype associated with poor clinical outcomes in colorectal cancer. *Nat Genet* (2024) doi:10.1038/s41588-024-01654-5.
2. Thorsson, V. *et al.* The Immune Landscape of Cancer. *Immunity* **48**, (2018).
3. Dunne, P. D. & Arends, M. J. Molecular pathological classification of colorectal cancer—an update. *Virchows Archiv* (2024) doi:10.1007/s00428-024-03746-3.
4. Reitsam, N. G. *et al.* Novel biomarker SARIFA in colorectal cancer: highly prognostic, not genetically driven and histologic indicator of a distinct tumor biology. *Cancer Gene Ther* **31**, 207–216 (2024).
5. Krogue, J. D. *et al.* Predicting lymph node metastasis from primary tumor histology and clinicopathologic factors in colorectal cancer using deep learning. *Communications Medicine* **3**, (2023).
