# Supplementary material for: Converging deep learning and human-observed tumor-adipocyte interaction as a biomarker in colorectal cancer
Source: Commun Med (Lond). 2024 Aug 15;4:163. doi: 10.1038/s43856-024-00589-6 (PMC11327259; doi:10.1038/s43856-024-00589-6)
Supplement: Supplementary file 3 — Description of Additional Supplementary Files [file 43856_2024_589_MOESM3_ESM.pdf]

Description of Additional Supplementary Files

File name- Supplementary Data 1

File description- Differing upregulated genes

File name- Supplementary Data 2

File description- Gene expression analysis

File name- Supplementary Data 3

File description- Clinical and molecular characteristics
